# Efficacy and safety of an improved Xiao-Xu-Ming-Tang traditional Chinese medicine hot compress for preventing catheter-related bladder discomfort: a multicenter, randomized, double-blind, controlled trial

**DOI:** 10.3389/fmed.2026.1799106

**Published:** 2026-04-22

**Authors:** Yujing Han, Zhihua Mi, Guanghui An, Bin Yang, Huixing Zhang, Dongmei Hong

**Affiliations:** 1 Department of Nursing, Shanghai General Hospital, Shanghai Jiao Tong University School of Medicine, Shanghai, China; 2 Shanghai Key Laboratory of Anesthesiology and Brain Functional Modulation, Department of Anesthesiology and Perioperative Medicine, Clinical Research Center for Anesthesiology and Perioperative Medicine, Translational Research Institute of Brain and Brain-Like Intelligence, Shanghai Fourth People’s Hospital, School of Medicine, Tongji University, Shanghai, China; 3 Department of Anesthesiology, Shanghai General Hospital, Shanghai Jiao Tong University School of Medicine, Shanghai, China; 4 Department of Anesthesiology, The First Affiliated Hospital of Xiamen University, Fujian, China

**Keywords:** catheter-related bladder discomfort, hot compress, improved Xiao-Xu-Ming-Tang, protocol, randomized controlled trial

## Abstract

**Objectives:**

Catheter-related bladder discomfort (CRBD) occurs in 47–95% of patients recovering from general anesthesia, causing discomfort and increasing the risk of postoperative delirium. Xiao-Xu-Ming-Tang is a traditional Chinese medicine (TCM) concoction that has *demonstrated efficacy* in relieving urinary symptoms. Thus, we propose the use of this improved traditional Chinese medicine concoction in the form of a hot compress as an inexpensive, effective, and simple intervention for preventing postoperative CRBD.

**Methods:**

This was a multicenter, randomized, double-blind, controlled study. Ninety patients who underwent transurethral ureteral laser lithotripsy at three centers were included. Patients were randomly divided into two groups: the Chinese medicine (CM) group and the control group (salt packet (SP) group), at a ratio of 1:1. In the CM group, a hot compress (40–45 °C) of improved Xiao-Xu-Ming-Tang mixed with salt was placed above the pubic symphysis immediately after intubation. In the control group, one-tenth of the improved Xiao-Xu-Ming-Tang and salt mixture was used.

**Results:**

The *primary endpoint* is the incidence of moderate or severe CRBD at 30 min after surgery. The secondary endpoints are the incidence of moderate-to-severe CRBD, the Numerical Rating Scale (NRS) Pain score, the incidence of skin discomfort above the pubic symphysis, and the vital signs (the blood pressure, heart rate, and oxygen saturation), and patient satisfaction after surgery.

**Discussion:**

The aim of this trial was to evaluate the effect and safety of a hot compress of an improved Xiao-Xu-Ming-Tang powder and salt mixture for moderate and severe CRBD. These findings may provide a basis for the prevention of CRBD in clinical practice.

**Clinical trial registration:**

http://itmctr.ccebtcm.org.cn/, Identifier: ITMCTR2025000321.

## 1 Introduction

Similar to overactive bladder, catheter-related bladder discomfort (CRBD) is mainly characterized by a frequent and urgent need to urinate as well as a burning sensation in the suprapubic region in patients recovering from anesthesia who have an indwelling catheter (1). CRBD may cause anxiety and pain, increase the need for anesthetics, and increase the incidence of postoperative complications (2). CRBD is not only a risk factor for postoperative agitation but also induces postoperative delirium in severe cases (3). Postoperative CRBD is a well-known side effect of indwelling catheter placement that occurs in 47%–95% of inpatients. Preventing or reducing the severity of CRBD is helpful for improving patients’ quality of recovery and reducing the incidence of postoperative agitation (4).

However, CRBD is difficult to prevent and treat. The main methods for preventing and treating CRBD include local anesthesia, psychological intervention and drug intervention. Currently, several methods have been proven to be effective for preventing CRBD, such as nerve block, transcutaneous electrical nerve stimulation, lidocaine, intravesical bupivacaine, magnesium sulfate, and nefopam (5–10). Because these methods are primarily implemented intraoperatively, the cost of the procedure increases. Thus, considering the substantial number of patients with indwelling catheters after surgery under general anesthesia, a cost-effective, feasible and effective strategy for preventing postoperative CRBD is needed. Traditional Chinese medicine (TCM) hot compresses have been used for more than 2,000 years. They can dilate local capillaries, improve blood circulation, reduce inflammation and swelling, dispel cold and dampness, alleviate pain and eliminate fatigue. TCM hot compresses offer a technically simple and cost-effective alternative (11, 12). TCM hot compresses can relieve pain and discomfort caused by muscle spasms as well as relieve breast tenderness and pain, post-stroke shoulder-hand syndrome and postpartum urinary retention (12–14). The improved Xiao-Xu-Ming-Tang powder is mixed with coarse sea salt (40–45 °C hot compress) to increase the skin permeability of the active components—the mild heat can dilate the suprapubic capillaries and open the skin stratum corneum pores, and the active compounds (volatile oils, alkaloids) of the medicinal materials are small molecular weight substances that can quickly penetrate the skin, enter the local subcutaneous tissue and bladder meridian, and exert anti-spasm and pain-relieving effects without systemic absorption (no systemic side effects) (15). Professor Peng Peichu, a renowned TCM expert in Shanghai, and his research team confirmed that Xiao-Xu-Ming-Tang can significantly alleviate catheter-induced bladder discomfort and irritation, as well as pain associated with urinary calculi (16). Based on clinical practice, our hospital’s TCM department optimized the original Xiao-Xu-Ming-Tang formula to form the improved version, which consists of *Notopterygii Rhizoma et Radix* (Qianghuo), *Chuanxiong Rhizoma* (Chuanxiong), *Scutellariae Radix* (Huangqin), *Ephedrae Herba* (Mahuang), *Aucklandiae Radix* (Muxiang), *Aconiti Radix Praeparata* (Zhi Fuzi), *Stephaniae Tetrandrae Radix* (Fangji), *Saposhnikoviae Radix* (Fangfeng), *Amomi Fructus* (Yangchun Sha), and *Paeoniae Radix Alba* (Sheng Baishao). The active components of these herbs have clear pharmacological effects on CRBD: *Notopterygii Rhizoma et Radix* contains volatile oils (e.g., ligustilide) and sesquiterpenoids that dilate local blood vessels and promote transdermal absorption, with its volatile components regulating the bladder meridian and relieving smooth muscle spasm; *Chuanxiong Rhizoma*, *Scutellariae Radix*, and *Ephedrae Herba* contain ephedrine (alkaloid) and volatile oils that inhibit M-cholinergic receptors of bladder smooth muscle and reduce involuntary contractions—the core pathological mechanism of CRBD—and their alkaloid components have good transdermal permeability, exerting local effects through the suprapubic skin; *Aucklandiae Radix* contains volatile oils (e.g., costunolide) for regulating qi and relieving spasm, with volatile components quickly penetrating the skin to act on local bladder meridians; *Amomi Fructus* contains volatile oils that soothe the bladder and relieve micturition urgency, with active components having high transdermal bioavailability on the lower abdominal skin. Most of these medicinal materials have a strong aromatic odor and good transdermal absorption properties; ground into powder and applied externally, they can regulate Zang-Fu organs and meridians to achieve the balance of yin and yang, which has been verified by relevant studies (17). CRBD falls under the categories of stranguria with urgency and abdominal pain in TCM, with its core pathogenesis being qi stagnation and meridian obstruction. Surgical trauma and catheter stimulation give rise to qi stagnation in the bladder meridian, which further induces bladder smooth muscle spasm. The modified Xiao-Xu-Ming-Tang is formulated based on the TCM therapeutic principles of regulating qi, unblocking meridians, and relieving spasm. The selected medicinal herbs target the bladder meridian in the lower abdomen, which accords with the standard TCM clinical practice of syndrome differentiation and treatment.

Our modification is dual-oriented: to adapt the formula for transdermal hot compress application and to achieve targeted relief of CRBD symptoms. (1) Component optimization: Based on the original prescription, Yangchun Sha and sheng Baishao were added to strengthen the actions of regulating qi, relieving spasm, and unblocking the bladder meridian, thereby exerting a targeted effect on CRBD caused by bladder smooth muscle spasm. Meanwhile, the dosages of Máhuáng and Fángjí were adjusted to reduce excessive pungency and ensure transdermal safety. (2) Formulation transformation: The original water decoction was converted into a powder mixture with coarse sea salt. The powder form increases the specific surface area of the medicinal materials, facilitating transdermal absorption. Coarse sea salt acts as a heat storage medium to maintain a stable temperature (40–45 °C) for hot compression, which is the key modification for adapting the prescription to transdermal hot compress application. (3) Efficacy re-orientation: The modified prescription abandons the systemic regulatory effect of oral administration and instead emphasizes local meridian regulation and transdermal delivery over the suprapubic region. It aims to directly alleviate bladder discomfort via the skin and meridians, which better matches the clinical demand for perioperative local intervention in CRBD.

Therefore, we hypothesized that improved traditional Chinese medicine hot compresses could reduce the incidence of moderate-to-severe CRBD in patients, providing a new method for preventing CRBD.

## 2 Methods and analysis

### 2.1 Study design

This is a multicenter, randomized, parallel group controlled study. The study period is November 2024–November 2025. A 24-h observation period will be included in the study (pictured). Patients who provide written informed consent will be tentatively enrolled and screened for eligibility during the observation period. In the experimental group, a hot compress (40–45 °C) of a Xiao-Xu-Ming-Tang and salt mixture will be placed above the pubic symphysis immediately after intubation. In the control group, a hot compress (40–45 °C) of one-tenth of an improved Xiao-Xu-Ming-Tang (This dose was confirmed to be non-therapeutic for CRBD based on our pre-experimental data and clinical dosage principles, cannot produce a significant effect on relieving bladder discomfort) and sea salt mixture will be placed above the pubic symphysis. During the observation period, the severity of CRBD will be graded using a scoring system developed and used by Agarwal et al. (18). The severity of CRBD will be divided into 4 grades: grade 1, the patient reports no symptoms of urinary tract or bladder discomfort; grade 2, the patient reports mild urinary tract or bladder discomfort only after prompting; grade 3, the patient reports moderate urinary tract or bladder discomfort without prompting but does not exhibit any behavior alluding to the discomfort; and grade 4, the patient reports severe urinary tract or bladder discomfort. The behaviors alluding to discomfort included flailing limbs, a strong vocal response, and attempts to remove the catheter.

*Experimental group (Composition of the TCM formulation)*: 9 g Qianghuo, 9 g Chuanxiong, 9 g Huangqin, 6 g Mahuang, 9 g Muxiang, 9 g Prepared *Aconitum Carmichaelii* Radix, 6 g Fangji, 9 g Fangfeng, 6 g Yangchun Sha, 9 g Sheng Baishao (full dose of improved Xiao-Xu-Ming-Tang) are ground into powder, mixed thoroughly with 100 g coarse sea salt, packed into a breathable cloth bag, heated to 40–45 °C, and applied as a hot compress above the pubic symphysis immediately after intubation until 60 min after surgery.

*Control group*: one-tenth of the full dose of improved Xiao-Xu-Ming-Tang powder (the proportion of each medicinal material is consistent with the experimental group), mixed thoroughly with 100 g coarse sea salt, packed into a breathable cloth bag, heated to 40–45 °C, and applied as a hot compress above the pubic symphysis immediately after intubation until 60 min after surgery.

*Supplemented the quality control details of the herbal powder*: A strict quality control system is implemented for the herbal powder:

*Preparation process quality control*: The grinding, mixing, and packaging processes are carried out in a GMP-compliant TCM preparation room; each batch of powder is sampled for active component content detection, and unqualified batches are discarded.

*Storage and transportation quality control*: The powder is stored in a sealed, dry, and cool environment (25 °C, relative humidity <60%); the hot compress bags are transported to each research center in a sealed carton, and the batch number and production date are marked to ensure traceability.

The study will be conducted in three general hospitals in China: Shanghai General Hospital, Shanghai Fourth People’s Hospital, and the First Affiliated Hospital of Xiamen University. The main research center is Shanghai General Hospital, and the principal investigator is Dongmei Hong. All evaluators will receive online centralized training and complete the assessment at each research center after passing the assessment.

### 2.2 Outcomes

#### 2.2.1 Primary outcomes

The primary outcomes will be the incidences of moderate and severe CRBD (grades 3 and 4) at 30 min after surgery.

#### 2.2.2 Secondary outcomes

The five secondary outcomes are as follows: (1) the incidence of moderate-to-severe CRBD at 1 h, 6 h, and 24 h after surgery; (2) the NRS score, which is used to evaluate postoperative pain, at 30 min, 1 h, 6 h, and 24 h after surgery, with scores ranging from 0, indicating no pain, to 10, indicating the worst pain imaginable; (3) the incidence of skin discomfort above the pubic symphysis (scald, redness, pruritus, allergy, etc.); (4) vital signs (blood pressure, heart rate, and oxygen saturation) at 10, 20, 30, 40, 50 min, 1 h, 6 h, and 24 h after surgery; and (5) patient satisfaction [24 h after surgery, patient satisfaction using a 5-point scale, with items graded 1 (“very dissatisfied”) to 5 (“very satisfied”)].

### 2.3 Participants

Patients who meet the inclusion criteria will be enrolled in the study. The inclusion criteria are as follows: (1) patients who are male sex and aged 18–45 years; (2) have a BMI less than 28 kg/m^2^; (3) are classified as ASA I-II; (4) undergo transurethral ureteral laser lithotripsy under quasi-general anesthesia, with an anesthesia time of 15–30 min; (5) have a no. 18 indwelling catheter after the operation; (6) are able to understand and willing to undergo evaluations related to CRBD as well as complete questionnaires; (7) sign the informed consent form voluntarily.

The exclusion criteria are as follows: (1) patients with serious heart, liver, kidney or other diseases; (2) patients with skin injuries, ulcers, sensory disturbances, acute closed injuries, suppurative infections or acute inflammation and other infectious diseases, skin diseases, severe diabetes, high fever with excessive product material at the superior margin of the pubic symphysis; and (3) patients with hypertrophy of the prostate, urethral stenosis, and a 1-year history of indwelling urethral catheters.

### 2.4 Randomization and masking

An envelope lottery will be used to randomize patients into groups, and because the study will be conducted across 3 centers, stratified randomization is required, with 30 patients included from each center, 15 of whom will be assigned to the experimental group and the remaining 15 will be included in the control group. The study will be conducted in double-blinded manner, and patients were only informed that they were enrolled in a clinical trial for the prevention of postoperative CRBD and were assigned to a study group with a unique study number, and the specific grouping (experimental/control) and the differences in the composition of the hot compress bags (full dose/one-tenth dose of medicinal powder) were not disclosed to the patients at any stage before, during, or after the surgery. The hot compress will be placed over the patient’s pubic symphysis immediately after intubation under general anesthesia and removed 60 min after surgery by a site nurse who will not be involved in follow-up visits. Patients, anesthesiologists, surgeons, note-takers, data registrars and analysts will be unaware of the group assignments. The TCM package will be distributed to each center by the Department of TCM of Shanghai Fourth People’s Hospital. Both groups will emit the smell of TCM, which are indistinguishable by odor alone. Group assignments will be maintained by the personnel at the main site who are not involved in the study but rather responsible for the study group numbers and hot compresses. Only when the patient has a serious adverse reaction that is suspected to be related to the intervention itself will he or she be responsible for the emergency unblinding of the patient to ensure timely treatment of the condition.

### 2.5 Patient and public involvement

Neither the patients nor the public will be involved in the design, implementation, reporting or planning of this study at any stage of the research process.

### 2.6 Sample size

The calculated sample size for this study is 90 patients: 45 patients in the experimental group and 45 patients in the control group. This is a randomized controlled study in which the experimental group will be treated with a TCM hot compress and the control group will be treated with a sea salt hot compress (a hot compress of a improved Xiao-Xu-Ming-Tang and salt mixture, making the two groups indistinct in smell) The primary outcomes will be the incidences of moderate and severe CRBD in patients with indwelling catheters after transurethral laser lithotripsy. According to previous studies, the incidence of moderate-to-severe CRBD after transurethral laser lithotripsy is 55–87% (3, 9, 20), with an average of approximately 70%. Assuming that the improved Xiao-Xu-Ming-Tang hot compress can reduce the incidence of CRBD by 50%, that is, the incidence of CRBD in the control group and the experimental group are 70 and 35%, respectively. Considering an *α* = 0.05 (two-tailed), a 1 − *β* = 0.9, and a 1:1 ratio, 42 patients are needed for the control group and 42 are needed for the experimental group. However, considering a loss to follow-up rate of 5%, approximately 45 patients will be included in each group.

### 2.7 Data management and statistical analysis

All the statistical analyses will be performed with SPSS version 27.0 (IBM Corporation, Armonk, NY, United States). The baseline data will include clinical data collected before the first intervention. Normally distributed continuous variables will be expressed as means ± standard deviations (SDs); otherwise, they will be expressed as medians and interquartile ranges (IQRs). Categorical variables will be described as numbers (*n*) and percentages (%). The chi-square test will be used to compare the incidence between the two groups, the t test will be used to compare normally distributed continuous variables, and the nonparametric test will be used for other variables. All hypothesis tests are two-sided, and two-sided *p*-values less than 0.05 represent statistical significance.

TSA 0.9.5.10 beta software will employed to verify study power, with preset parameters including two-sided *α* = 0.05, *β* = 0.1, and an expected RRR of 50%. Using the primary outcome (incidence of moderate–severe CRBD), we will derive the cumulative *Z*-value and establish the monitoring boundary to guard against false positives and false negatives induced by multiple testing.

For NNT reporting, we calculate and report the NNT (95% CI) for the primary endpoint (30-min post-surgery moderate–severe CRBD) in the Results section. Defined as 1/ARR, the NNT directly quantifies the clinical impact of the optimized Xiao-Xu-Ming-Tang hot compress, representing the number of subjects needed to prevent one instance of moderate–severe CRBD.

### 2.8 Study procedures and interventions

**Figure 1:**
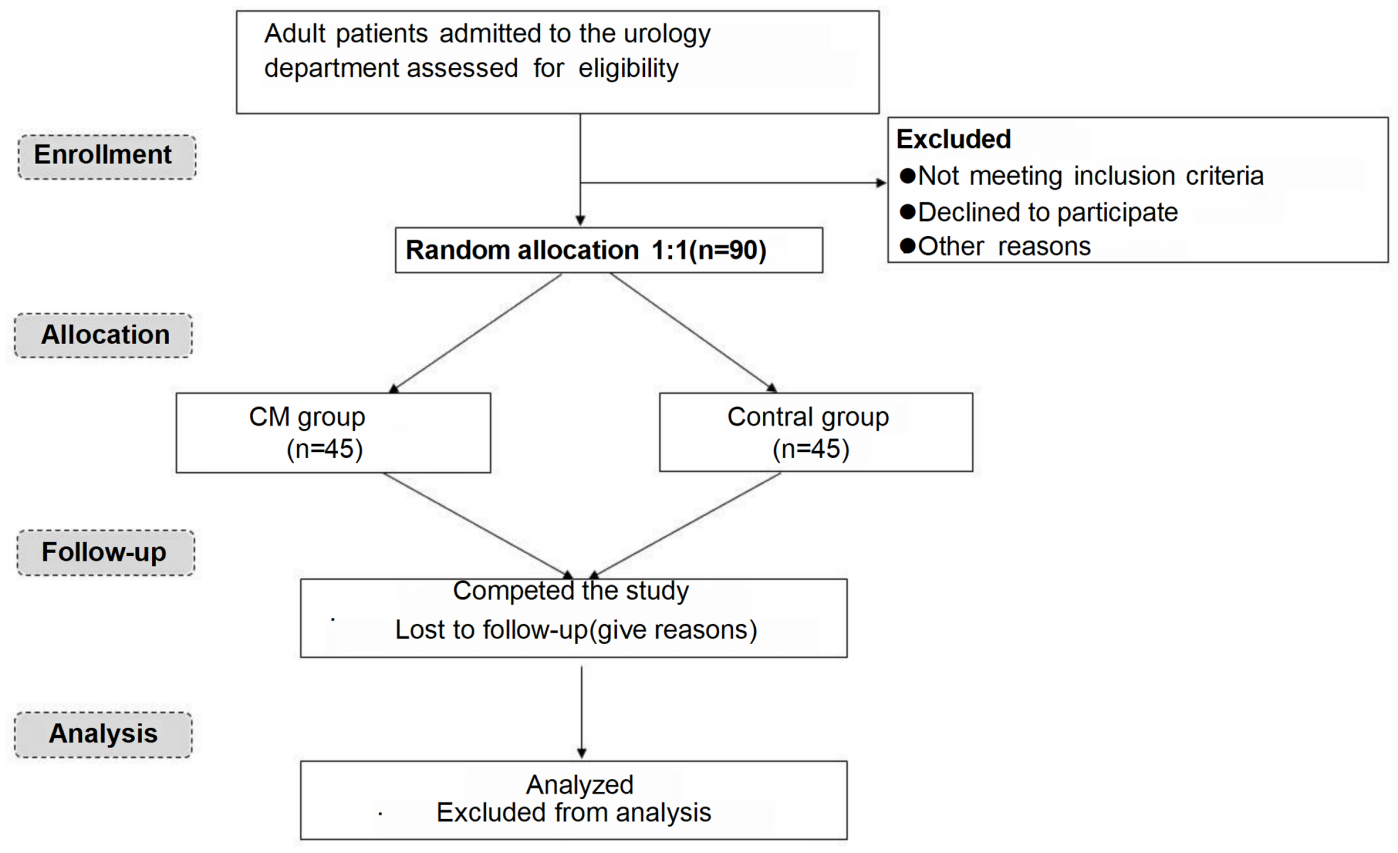
Flow chart of patients.

This study will be divided into three periods: a screening period, a treatment period and a follow-up period. The flow chart of this trial is presented in Figure 1.

#### 2.8.1 Screening period: admission to the hospital to the start of surgery

After admission, the patients will undergo an examination and sign an informed consent form after screening. Patients will be instructed on the use of the NRS and CRBD evaluating prior to surgery to avoid confusion.

#### 2.8.2 Treatment period: start of surgery to 1 h after the end of surgery

Anesthesia protocol (all procedures will be performed under standardized anesthesia): Patients will be admitted to the operating room without pre-anesthesia medication and undergo electrocardiogram (ECG), pulse oxygen saturation (SpO_2_), blood pressure, and depth of anesthesia (BIS or Narcotrend) monitoring. Five hundred milliliters of Ringer’s solution will be given through peripheral venous access. Sufentanil (0.1–0.3 μg/kg) and cyclopofol (0.4–0.6 mg/kg) will be used for the induction of general anesthesia. A muscle relaxant (rocuronium 0.6 mg/kg) will be given, and a laryngeal mask will be placed when the depth of anesthesia is deemed adequate (a BIS of 45–60). After anesthesia induction, a hot compress will be placed over the patient’s pubic symphysis immediately by a research nurse (to avoid a burn injury, the temperature of the hot compress should remain between 40 and 45 °C during continuous monitoring at the contact surface with a temperature probe. The surgery will be performed as usual. The injection rates of propofol and remifentanil will be adjusted according to depth of anesthesia and the patient’s hemodynamics during the operation, and the depth of anesthesia must be maintained at 45–60. The volumetric ventilation mode will be adopted with a tidal volume set at 6–8 mL/kg, an airway pressure maintained at 30 cmH_2_O, and the ETCO_2_ maintained at 35–45 mmHg. The patient’s ECG, HR, BP, RR, SpO_2_ and EtCO_2_ will be monitored intraoperatively. At the end of surgery, the anesthesiologist will discontinue infusion of all anesthetics and then administer sugammadex (2 mg/kg). A no. 18 indwelling catheter will be placed by the urologist, and a balloon will be expanded with 10 mL of distilled water. The laryngeal mask will be removed when the patient can open his/her eyes when prompted, and he/she will be transferred to the postoperative recovery room (PACU) for observation for at least 60 min before being transferred back to the ward. All cases of moderate or severe CRBD during resuscitation will be managed and recorded by the resuscitation room physician.

#### 2.8.3 Follow-up period: end of surgery to 24 h after surgery

NRS and RASS scores will be evaluated by a dedicated follow-up researcher at 30 min, 1 h, 6 h, and 24 h after surgery. CRBD grades and patient satisfaction scores will be recorded at the same postoperative time points. The researcher responsible for documentation will complete the CRF. Patients’ vital signs on admission to the PACU, intraoperative anesthetic agents, RASS, NRS, CRBD, and adverse events will be recorded in the electronic data system. The study timeline is presented in Table 1.

**Table 1:** Timetable of the study period.

Timepoint	Screening period (−2~−1 d)	Preparation period (−1~0 d)	Surgery period (0 d)	Follow-up period (after awakening until 24 h postoperatively)								
		T0		T1	T2	T3	T4	T5	T6	T7	T8	T9
Eligibility screen and informed consent	X											
General condition of the patient^a^	X											
Training^b^		X										
Vital signs^c^	X			X	X	X	X	X		X	X	X
Random allocation		X										
Hot compress			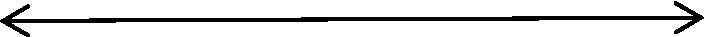									
The CRBD score						X				X	X	X
The NRS score						X				X	X	X
Adverse events record^d^			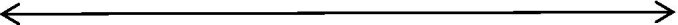									
Satisfaction of patients												

*T0, Before induction of anesthesia; T1, 10 min after awakening; T2, 20 min after awakening; T3, 30 min after awakening; T4, 40 min after awakening; T5, 50 min after awakening; T6, 1 h after awakening; T7, 6 h after awakening; T8, 12 h after awakening; T9, 24 h after awakening.

a Patients were educated about how to recognize CRBD (for example, suprapubic discomfort or burning with the urge to urinate) and how to distinguish them from surgical pain.

b Obtain the patient’s name, hospitalization number, age, medical history (whether there are the following conditions, serious heart, liver, kidney and other diseases; If the hot compress site has skin injury, ulcer, sensory disturbance, acute closed injury, suppurative infection or acute inflammation and other infectious diseases, skin diseases, severe diabetes, high fever and excessive use of product materials, it is not suitable for hot compress; Patients with prostatic hypertrophy, urethral stricture, and a history of indwelling catheter within 1 year.

c Blood pressure, heart rate, and oxygen saturation.

d Burns, redness, itching, allergies, etc.

To ensure strict compliance with the measurement time points, the study procedures were optimized. The follow-up researcher will perform assessments at the ward or PACU exactly at the scheduled times. Vital signs will be documented by nurses at fixed time points, and all data will be entered into the electronic system with time stamps to guarantee accuracy and consistency. All researchers and nurses received uniform training to standardize assessment procedures and minimize human-related time-point deviations.

## 3 Discussion

In this multicenter RCT, we will evaluate the effectiveness of an improved version of the TCM hot compress of improved Xiao-Xu-Ming-Tang in safely alleviating CRBD after surgery for ureteral calculi. This study will provide evidence of the efficacy of this innovative intervention in relieving CRBD and therefore improving patient satisfaction.

CRBD is highly prevalent worldwide (47%–95%). Its pathophysiology-M-cholinergic receptor-mediated involuntary contraction of bladder smooth muscle is consistent across populations (4). CRBD is mainly characterized by discomfort in the suprapubic region and a frequent and urgent need to urinate, with or without urinary incontinence. In the early stage of recovery from general anesthesia, patients are not fully conscious, are disoriented, have increased pain sensitivity, and are prone to CRBD (1, 19). But the core drawbacks of traditional preventive methods for CRBD lie in three key dimensions: limited efficacy, significant side effects, and poor compliance. Furthermore, most of these methods only address symptoms rather than the root causes, making it difficult to eradicate the risk fundamentally (20).

This discomfort is believed to be induced by involuntary bladder smooth muscle contractions mediated by muscarinic receptors. Medications such as ketamine, nefopam, dexmedetomidine, and tramadol are commonly used to manage CRBD (21–24). Additionally, transcutaneous electrical nerve stimulation, acupuncture, and pudendal block have also been reported to be effective in the prevention and treatment of CRBD (6, 25, 26). However, these management strategies can have various adverse effects, such as postoperative nausea and vomiting, dry mouth, headache, facial flushing, hypotension, blurred vision, seizure, muscle weakness, and pelvic floor muscle weakness. TCM is expected to prevent postoperative CRBD because of its safety, easy application and repeatability. Given its excellent safety profile, ease of administration, and reproducibility, TCM holds significant promise for preventing postoperative CRBD. Its implementation does not rely on intricate cultural or medical constructs, thereby facilitating its global adoption across diverse healthcare systems and regions. Furthermore, the hot compress procedure is highly standardized (with fixed temperature, site, and duration), enabling rapid proficiency among medical professionals from various professional backgrounds (15). In this study, the intervention will continue from the time of anesthesia induction to ensure a sustained effect. It can effectively reduce the incidence of complications after general anesthesia, shorten the length of PACU stay, and reduce the length of hospital stay.

Some limitations of this study should also be considered. Firstly, because the main outcome of this study is the incidence of moderate to severe CRBD, which is the subjective feeling of patients. To minimize confounding variables of patient factors on the trial, we strictly restricted the gender, age, type of surgery, and duration of anesthesia. Secondly, urethral catheterization was performed with a standard 18-Fr Foley catheter, thus comparisons of other sized catheters were not possible. Large-diameter Foley catheters are an independent risk factor for CRBD. In addition, we did not perform urine flow assays, residual urine analyses, or prostate size evaluations, possibly confounding the results.

## 4 Conclusion

This multicenter, randomized, double-blind, controlled trial will provide high-quality clinical evidence on whether the improved Xiao-Xu-Ming-Tang traditional Chinese medicine hot compress offers a safe, non-invasive, and cost-effective strategy for preventing postoperative moderate–severe CRBD in patients undergoing transurethral ureteral laser lithotripsy under general anesthesia. If the intervention is proven effective, it will provide a new clinical option for the prevention of CRBD, especially for clinical settings that require low-cost and easy-to-operate interventions, and has the potential for wide clinical promotion worldwide.
